# Towards a Physiology-Based Measure of Pain: Patterns of Human Brain Activity Distinguish Painful from Non-Painful Thermal Stimulation

**DOI:** 10.1371/journal.pone.0024124

**Published:** 2011-09-13

**Authors:** Justin E. Brown, Neil Chatterjee, Jarred Younger, Sean Mackey

**Affiliations:** 1 Department of Anesthesia, Stanford University, Palo Alto, California, United States of America; 2 Neurosciences Program, Stanford University, Palo Alto, California, United States of America; 3 Department of Biology and Environmental Science, Simpson College, Indianola, Iowa, United States of America; 4 Feinberg School of Medicine, Northwestern University, Chicago, Illinois, United States of America; City of Hope, United States of America

## Abstract

Pain often exists in the absence of observable injury; therefore, the gold standard for pain assessment has long been self-report. Because the inability to verbally communicate can prevent effective pain management, research efforts have focused on the development of a tool that accurately assesses pain without depending on self-report. Those previous efforts have not proven successful at substituting self-report with a clinically valid, physiology-based measure of pain. Recent neuroimaging data suggest that functional magnetic resonance imaging (fMRI) and support vector machine (SVM) learning can be jointly used to accurately assess cognitive states. Therefore, we hypothesized that an SVM trained on fMRI data can assess pain in the absence of self-report. In fMRI experiments, 24 individuals were presented painful and nonpainful thermal stimuli. Using eight individuals, we trained a linear SVM to distinguish these stimuli using whole-brain patterns of activity. We assessed the performance of this trained SVM model by testing it on 16 individuals whose data were not used for training. The whole-brain SVM was 81% accurate at distinguishing painful from non-painful stimuli (*p*<0.0000001). Using distance from the SVM hyperplane as a confidence measure, accuracy was further increased to 84%, albeit at the expense of excluding 15% of the stimuli that were the most difficult to classify. Overall performance of the SVM was primarily affected by activity in pain-processing regions of the brain including the primary somatosensory cortex, secondary somatosensory cortex, insular cortex, primary motor cortex, and cingulate cortex. Region of interest (ROI) analyses revealed that whole-brain patterns of activity led to more accurate classification than localized activity from individual brain regions. Our findings demonstrate that fMRI with SVM learning can assess pain without requiring any communication from the person being tested. We outline tasks that should be completed to advance this approach toward use in clinical settings.

## Introduction

Pain is commonly accepted to be a subjective experience [1], for which the gold standard of measurement is self-report. While self-reported pain provides useful clinical information and proves to be an effective assessment approach in most situations, it can fail certain vulnerable populations. Individuals with major cognitive or communicative impairments, such as intensive care unit patients or older adults with dementia, may not be able to provide valid self-reports of pain [2], [3]. For those individuals, there are few methods for determining the presence or absence of pain. While behavioral tools exist (such as those assessing facial expressions, vocalizations, and body movements) [4]–[6], they too may fail individuals with paralyses or other disorders affecting motor behavior. There is, therefore, a need to develop a pain assessment tool that is based on physiology, and requires no communication on the part of patients.

Researchers have long sought to develop a physiology-based pain assessment that does not depend on patient volitional behaviors [7]. Those efforts have focused on various biosignals, such as heart rate [8], [9], skin conductance [10], and electroencephalography [11]. While several physiologic variables have shown statistically significant correlations with the presence of pain, or with pain intensity, no measure has provided a sufficiently high relationship with pain to be used as a valid surrogate for self-reports [12]–[14]. Therefore, despite many years of research, there is currently no accepted technique for the physiologic assessment of pain in humans.

Recent advances in neuroimaging have provided possibilities for pain assessment that have not traditionally been available to researchers [15]. By measuring physiologic events that are closely associated with neural activity, noninvasive neuroimaging methods such as functional magnetic resonance imaging (fMRI) gain an advantage over previously employed methods of physiologic pain assessment. The use of fMRI in detecting the presence of pain may be particularly strengthened by incorporating machine learning algorithms. Machine learning algorithms, such as the support vector machine (SVM), can allow predictive models to be trained with a known set of stimuli, and then used to classify novel stimuli. An SVM can be trained on patterns of whole-brain activity, in order to find the linear combinations of regional brain activity that best distinguishes two experiential states. Using this approach, machine learning algorithms have recently been used, in conjunction with fMRI data, to determine what a person is seeing or hearing [16], [17]. Properly developed, the union of fMRI and SVM may provide a valid, physiology-based proxy for self-reported pain.

Marquand and colleagues (2010) were the first to apply fMRI and machine learning algorithms to the problem of pain measurement [18]. In their study, healthy individuals were exposed to thermal stimuli presented at heat perception threshold, pain perception threshold, and pain tolerance. Machine learning algorithms were trained on fMRI data and used to predict self-reported pain for each participant individually (i.e., one model per participant). Each individual's model was then used to classify subsequent stimuli in that same individual. The SVM model was reported to have a classification accuracy that ranged from 68.34% (distinguishing pain detection from pain tolerance) to 91.67% (distinguishing heat threshold from pain tolerance). This study provided an important advancement in pain measurement, demonstrating that machine learning algorithms could be used to assess an individual's pain, if trained using fMRI data from that same individual.

An important extension of the work of Marquand et al. would be to demonstrate that physiology-based pain assessment, using fMRI data and machine learning algorithms, can classify pain accurately without relying on self-report data from the individual tested. If, for example, an SVM model could be trained on one set of individuals, and used to accurately classify pain in different individuals, then its performance would not depend on the test subjects' self-report.

In this study, we attempted to develop an SVM model that accurately determines the presence or absence of pain, even when tested on individuals whose self-reported data were not included in the model's training. Towards this aim, we investigated the task of distinguishing non-painful heat stimuli from painful heat stimuli. The major goal of the study was to determine whether blood-oxygen-level dependent (BOLD) signal change is sufficiently consistent between individuals to potentially train a physiology-based pain classifier that performs accurately when trained on one group of subjects and tested on another. An SVM model was trained on a group of eight individuals, and used to classify pain in a separate group of eight individuals. When tested on this separate group of eight individual, the SVM was significantly more accurate than chance. In a second study, the same SVM model was further validated through test-retest reliability in an additional group of eight individuals. When tested on this additional group of eight individuals, the SVM was again significantly more accurate than chance.

## Methods

### Study 1: Training and Initial Validation of the SVM Model

#### Participants

Nineteen participants were recruited via advertisements posted on and around the Stanford University campus. All participants were healthy and none reported having a chronic pain condition. Procedures were approved by the Stanford University School of Medicine Institutional Review Board, and all participants provided written informed consent. Due to technical difficulties with the temperature thresholding or scan procedures, complete data were not collected for three participants; therefore, they were excluded from all analyses. The remaining 16 participants were an average age of 22.7 years (SD = 3.6), with 10 men and 6 women.

#### Protocol

Before starting the fMRI scanning session, participants were thresholded with a thermal stimulator in order to determine individual temperatures for painful heat. Thermal stimuli were delivered to the left volar forearm via a 3×3 cm Peltier-type thermode (Medoc, North Carolina). A range of temperatures was presented, each for 30 seconds. Following each temperature presentation, the participant provided a self-report of pain on a 0–10 numerical rating scale with the following anchors: 0 (no pain), 3 (minor pain), 5 (moderate pain), 7 (intense pain that you can bear without moving), and 10 (unbearable pain). The thresholding procedures used here have been previously described in greater detail [19]. The temperature that consistently elicited a 7 out of 10 pain score was used as the painful stimulus temperature in the fMRI scanning session. The average temperature selected for painful stimulation was 46.3°C (SD = 1.1).

During the fMRI sessions, heat stimuli were again presented to the left volar forearm in a block design, with 40 seconds of baseline temperature (at 26°C), followed by 30 seconds of heat stimulation. All participants completed four functional runs. In two runs, participants received hot but non-painful heat stimulation (38°C). In the other two runs, participants received painful heat stimulation (individually calibrated to elicit a pain score of seven). Each participant received a total of 14 nonpainful stimuli and 14 painful stimuli. Following each functional run, participants reported whether the stimuli presented were painful or non-painful.

#### MRI Data Collection and Standard Pre-Processing

FMRI data were collected on a 3.0 Tesla, whole-body scanner (GE Healthcare Discovery 750), using an 8-channel receive-only phased-array head coil. A T1-weighted fast spoiled gradient-recall scan was acquired for anatomical reference (TE = 2.0 ms, 156 slices at 1.3 mm thickness). High-order shimming [20] was then performed, followed by the functional runs. Functional imaging used a T2*-sensitive gradient spiral in/out pulse sequence [21], with a TR of 2000 ms, TE = 20 ms, flip angle = 77°, 64×64 acquisition matrix, and 30, 4 mm interleaved slices parallel to the intercommissural line. Images were corrected for cardiac and respiratory noise using RETROICOR [22]. SPM5 (Wellcome Trust Centre) was used for functional image realignment and motion correction, coregistration to the structural images, and normalization to the Montreal Neurologic Institute (MNI) stereotactic template. As a final processing step, functional images were spatially smoothed with a 3D Gaussian kernel (4 mm full-width half-maximum).

#### SVM Model Pre-Processing

SVM model pre-processing was conducted in MATLAB (Mathworks) using SPM5 and custom software. A whole-brain pattern of the activity induced by each heat stimulus was computed as map of percent BOLD signal change. For each heat stimulus, the average percent BOLD change was calculated with the following formula: ((average stimulus signal – average baseline signal)/average baseline signal). The baseline signal consisted of the 20 seconds before each heat stimulus. The stimulus signal consisted of the final 24 seconds of each heat block, excluding the initial 6 seconds to allow for the BOLD signal to reach its peak intensity. Each of the maps of percent BOLD signal change constitutes an *example* to be used for training and testing the SVM. In this way, by treating each heat stimulus as a single example, the SVM preprocessing resulted in 448 examples: 28 heat stimuli ×16 subjects. Each of these examples was a map of percent BOLD change containing 18,124,575 features (voxels).

Feature reduction (to avoid over-fitting of the model) was achieved by applying a gray matter mask to exclude areas that did not containing neuron cell bodies. Typically, the magnitude of pain-induced BOLD signal change is less than 1% [23]–[27]. Thus, an additional and liberal feature reduction step was achieved by excluding any voxel exhibiting percent signal change greater than 3%, on the grounds that large changes are likely artifactual. The feature reduction steps reduced the number of features in each example from 18,124,575 to 65,839, with each feature corresponding to average percent BOLD change in a single gray-matter voxel.

#### SVM Model Training

Eight of the sixteen participants were randomly assigned to the model training group. Randomization was performed using a computer-based list randomizer. As described previously [28], the SVM model training was performed using a multi-voxel pattern analysis approach, conducted in MATLAB using an SVM toolbox written by Anton Schwaighofer (mail to: anton.schwaighofer@gmx.net). Using examples of painful and non-painful heat stimuli from the eight training group participants, a linear SVM was trained to classify heat stimuli as painful or non-painful (the regularization parameter, C, was set at 10 prior to training). Using a linear combination of the features (the magnitude of percent BOLD change in each voxel), the SVM determined a function to best predict whether each example (each heat stimulus) in the training set was painful or non-painful. Numerically, this function is of the form: Y = W_1_X_1_+W_2_X_2_ …+W_N_X_N_+Z, where for each example, N is the number of features (number of voxels), W is the weighting of each feature, X is the value of each feature (the percent BOLD signal change), and Z is a constant. If Y is positive, then the example is classified as painful, and if Y is negative, then the example is classified as nonpainful. The function used for SVM classification is often described visually: each example (each map of BOLD signal) can be thought of as a point in space, and the SVM can be thought of as determining the separating hyperplane which best separates those points in space associated with painful stimulation from those associated with nonpainful stimulation. For a more detailed discussion of the mathematical method, see C. Cortes and V. Vapnik (1995) [29].

#### SVM Model Testing

The trained SVM model was then used to classify pain in the eight individuals who were randomly assigned to the testing group. For each heat stimulus presented to participants in the testing group, the SVM model assigned a classification of painful or non-painful. The SVM also calculated a measure of confidence in the accuracy of each assignment. This measure of confidence was derived from the distance of each example (each map of percent BOLD change) from the separating hyperplane. The percent of accurate classifications was calculated for each participant in the testing group, as well as positive predictive value (PPV) and negative predictive value (NPV). PPV is the percent of stimuli predicted to be painful which were actually painful, while NPV is the percent of stimuli predicted to be non-painful which were actually non-painful.

#### Permutation Testing

To identify which brain regions significantly influenced the SVM classifier's accuracy, we conducted a permutation test as previously described [28]. In brief, we tested the null hypothesis that patterns of brain activity did not influence the performance of the SVM classifier. To derive the null distribution by permutation, the class labels (painful or non-painful) were randomized 750 times. With each randomization, a linear SVM was trained to distinguish the examples (the maps of percent BOLD change) that were randomly labeled painful from the examples that were randomly labeled non-painful. To determine statistical significance, the model resulting from accurate class labeling was compared to the empirically derived null distribution. By randomly rearranging the training data, this permutation test reveals which brain regions significantly affected the training of the SVM, and thus contributed to the SVM classifier's performance at distinguishing painful from non-painful stimulation.

#### Region of Interest Analysis

To test whether any regions might independently distinguish painful and non-painful stimuli, we conducted individual SVM classifications using small regions of interest (ROIs). Based on a meta-analysis of 68 studies, Apkarian et al. (2005) have proposed that there is a brain network for acute pain which is composed of 6 brain regions: the primary and secondary somatosensory cortices, the insular cortex, the anterior cingulate cortex, the prefrontal cortex, and the thalamus [30]. In separate SVM analyses, we investigated each of those six areas. Each ROI was functionally defined as an 8 mm sphere centered on previously reported coordinates which were identified in a study of pain processing [31]. For each ROI, the average percent BOLD signal change was computed for each heat stimulus. These measurements of percent BOLD signal change constitute the examples used to train and test an SVM for each ROI. As with the whole-brain SVM analysis, each ROI classifier was trained on fMRI data from the training group (N = 8) and tested on fMRI data from the testing group (N = 8).

### Study 2: Confirmatory Validation of the SVM Model

In order to provide an additional validation of the SVM model by test-retest reliability, an additional nine participants were recruited and assigned to an independent retesting group. As with the previous groups, participants were recruited from Stanford and from the surrounding area. Due to technical difficulties with the temperature thresholding procedures, complete data were not collected for one participant; therefore, this participant's data were excluded from all analyses. The remaining eight participants were an average age of 25.9 years (SD = 3.3), with 5 men and 3 women. Participants in Study 2 followed the same procedures as those participants in Study 1 (with the exception that no randomization was conducted because all participants were assigned to a single retest group). The average temperature for the painful stimulation was 46.0°C (SD = 1.0). The SVM model trained in Study 1 was used to classify painful and non-painful stimuli in the participants recruited to Study 2.

## Results

### Study 1: Training and Initial Validation of the SVM Model

As a validity check on the effectiveness of the chosen temperatures to elicit painful and non-painful sensations, we first examined self-reported pain. All included participants reported that the experimental temperature that was thresholded to a 7 out of 10 pain score elicited pain, and that the 38°C temperature did not.

The SVM model, which was trained on data from participants in the training group, performed significantly better than chance when distinguishing painful from non-painful stimuli in participants from the independent testing group (*t* (7) = 9.9, *p* = 0.00002). Average accuracy was 86.6%. As seen in Table 1, accuracy ranged from 71.4% to 100% across the eight testing individuals. Average PPV (stimuli classified as painful which were actually painful) was 90.3%, and NPV (stimuli classified as non-painful which were actually non-painful) was 85.4%.

**Table 1:** Performance of the Whole-Brain SVM Classifier.

Participant	Accuracy (%)	Positive PV (%)	Negative PV (%)
1	75.0	100	66.7
2	85.7	91.7	81.2
3	82.1	80.0	84.6
4	100.0	100.0	100
5	71.4	71.4	71.4
6	96.4	93.3	100
7	85.7	85.7	85.7
8	96.4	100	93.3
Average	86.6±10.4*	90.3±10.5*	85.4±12.3*

For each participant in the testing group, the SVM was used to distinguish the painful stimuli from the nonpainful stimuli. For each participant in the testing group, and for their group average, this table displays the SVM's overall accuracy, and positive and negative predictive value (PV). Error is reported as 1 standard deviation. An asterisk indicates performance measures that are significantly greater than chance (*p<0.*05).

**Figure 1:**
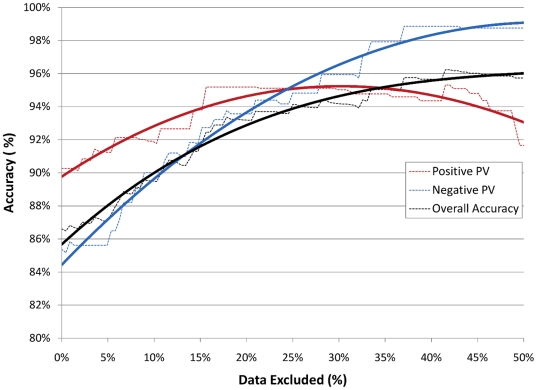
Performance of the Whole-Brain SVM Classifier is Increased by a Distance Threshold. The classifier's performance was assessed at increasing distance thresholds. As the distance threshold increased, an increasing number of stimuli were excluded on the grounds that stimuli nearest the separating hyperplane were most likely misclassified. In this figure, performance is plotted as a function of the percentage of stimuli that have been excluded from classification. Dotted lines display the performance computed at each distance threshold. Solid lines display a third degree polynomial fit to those data.

We next examined whether classification accuracy could be improved by incorporating a confidence threshold, measured as distance from the separating hyperplane. BOLD maps not meeting the confidence threshold were excluded on the basis of insufficient evidence to make a confident classification. Overall accuracy of the SVM classifier increased monotonically with the number of stimuli excluded (Figure 1). When excluding the 15% of stimuli that were nearest to the hyperplane, the best balance was achieved between maximizing the accuracy of the SVM classifier and minimizing the number of excluded stimuli. Specifically, when excluding the 15% of stimuli that were nearest to the hyperplane, overall accuracy was increased to 91.8%, PPV was increased to 93.8% and NPV was increased to 92.7% (Figure 1).

**Figure 2:**
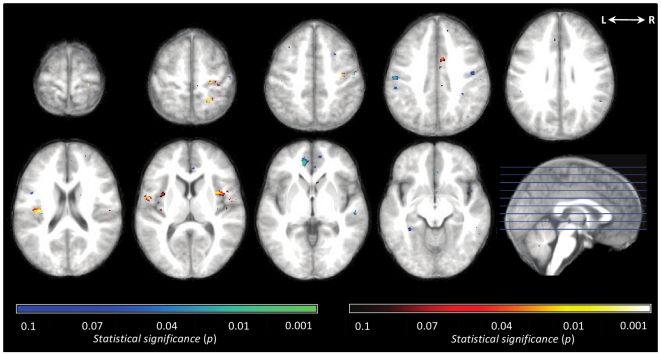
Brain Regions that Most Influenced the Whole-Brain SVM Classifier. A permutation test was run to determine which brain regions significantly affected the whole-brain SVM classification. This figure illustrates brain regions that fall within the 90^th^ percentile of the null distribution that was determined by permutation. Regions in the 99^th^ percentile (*p<0.01)* are noted in the results section. Shades of red indicate regions where greater BOLD signal influenced the SVM to classify a stimulus as painful. Shades of blue indicate regions where greater BOLD signal influenced the SVM to classify a stimulus as non-painful.

Next, a permutation test was used to determine which brain regions were most involved in driving the whole-brain SVM classifier's performance. The classification of a stimulus as painful was significantly influenced (*p<0.*01) by greater BOLD signal in the bilateral mid-insular cortices (MNI: −36, 0, 12 and 36, 4, 12), bilateral secondary somatosensory cortices (−34, −18, 16 and 38, −18, 20), contralateral posterior insular cortex (36, −16, 14), contralateral primary somatosensory cortex (22, −26, 54 and 24, −46, 6), and contralateral primary motor cortex (36, −14, 48). The classification of a stimulus as nonpainful was significantly influenced (*p<0.01*) by greater BOLD signal in the bilateral primary motor cortices (−44, −12, 36 and 52, −14, 48), and ipsilateral pregenual cingulate cortex (−10, 42, 2). Those brain regions, which significantly affected the whole-brain SVM classification, are illustrated in Figure 2.

Because using information from a single ROI to assess pain would be simpler than employing a whole-brain SVM, we next tested whether BOLD signal from individual ROI's could distinguish painful and non-painful stimuli as accurately as the whole-brain SVM model. Activity in the secondary somatosensory cortex classified painful stimuli significantly better than chance (*t* (7) = 5.0, *p* = 0.0016), as did activity in the mid-insular cortex (*t* (7) = 4.0, *p* = 0.0052). As seen in Table 2, for the other regions tested, overall accuracy did not reach the *p*<0.05 level of significance for classifying stimuli as painful or non-painful. Classification based on activity in the secondary somatosensory cortex (71.9%) and mid-insular cortex (64.3%), did not reach the same level of accuracy as classification based on whole-brain patterns of activity (86.6%).

**Table 2:** Performance of the ROI SVM Classifiers.

Region	Accuracy (%)	Positive PV (%)	Negative PV (%)
ACC	56.7±12.1	57.7±17.5	57.1±11.1
Insula	64.3±10.1*	66.2±11.8*	63.3±9.1*
PFC	50.0±12.2	46.2±16.9	53.2±12.7
S1	54.0±15.7	46.2±30.7	556.3±16.2
S2	71.9±12.4*	75.2±13.9*	71.2±13.1*
Thalamus	59.8±11.9	58.0±14.7	61.8±12.1*

Using the activity from six regions of interest (ROIs), SVMs were used to distinguish the painful stimuli from the nonpainful stimuli. For each ROI, this table displays the SVM's average accuracy, and positive and negative predictive value (PV) when tested on participants in the testing group (N = 8). Error is reported as 1 standard deviation. An asterisk indicates performance measures that are significantly greater than chance (*p<0.*05). Anterior cingulate cortex (ACC). Primary somatosensory cortex (S1). Secondary somatosensory cortex (S2). Prefrontal cortex (PFC).

### Study 2: Confirmatory Validation of the SVM Model

An additional group of eight participants was investigated to determine test-retest reliability of the SVM model in classifying painful stimuli. As seen in Table 3, accuracy in this second group was 74.6% (*t (7)* = 5.5, *p* = 0.0009). Average PPV was 83.6% and average NPV was 74.4%. We again examined whether accuracy could be improved by incorporating a confidence threshold based on distance from the separating hyperplane. In the retest group, as with the initial test group, when excluding the 15% of stimuli that were nearest to the hyperplane, the performance of the SVM classifier improved. The exact value of the confidence threshold differed between test groups, so that in both analyses, 15% of the stimuli were excluded. When excluding 15% of stimuli from the retest group, the overall accuracy was increased to 76.9%, PPV was increased to 85.8%, and NPV was increased to 76.7%.

**Table 3:** Retest Validation of Performance of the Whole-Brain SVM Classifier.

Participant	Accuracy (%)	Positive PV (%)	Negative PV (%)
1	64.3	66.7	62.5
2	85.7	77.8	100.0
3	64.3	70.0	61.1
4	71.4	100.0	63.6
5	67.9	66.7	69.2
6	92.9	87.5	100.0
7	60.7	100.0	56.0
8	89.3	100.0	82.4
Average	74.6±12.7*	83.6±15.2*	74.4±17.6*

For each participant in the re-testing group, the SVM from study 1 was used to distinguish the painful stimuli from the nonpainful stimuli. For each participant in the retesting group, and for their group average, this table displays the SVM's overall accuracy, and positive and negative predictive value (PV). Error is reported as 1 standard deviation. An asterisk indicates performance measures that are significantly greater than chance (*p<0.*05).

## Discussion

In this study, we establish the feasibility of physiology-based pain detection using BOLD fMRI data and supervised machine learning algorithms. An SVM model, trained on 8 individuals, was 80.6% accurate at distinguishing painful from non-painful stimuli when tested on 16 individuals whose self-report data were not used in training.

BOLD activity in five brain regions was principally responsible for the SVM classifier's performance at distinguishing painful from non-painful stimulation. Increased activity in the primary somatosensory cortex, secondary somatosensory cortex, insular cortex, and primary motor cortex was predictive of painful stimulation. Increased activity in other areas of the primary motor cortex and in the pregenual anterior cingulate cortex was predicative of nonpainful stimulation. These five areas are consistent with prior literature that identifies critical pain processing regions of the human brain [30], [32], [33]. The primary sensory cortex, secondary sensory cortex, and insular cortex have been implicated in sensory aspects of pain perception, the insular cortex has been implicated in emotional aspects of pain perception, and the primary motor cortex may play a role in the inhibition of reflexive withdraw from pain. Increased activity in the pregenual anterior cingulate cortex has been implicated in happiness [34]; we speculate that pain may have caused a state of unhappiness leading to decreased activity in this region.

Because the SVM model was powered by a relatively small set of brain regions, we were interested to know if activity in any one brain region could classify pain equally as well as the whole-brain approach. When tested, we found that an SVM, using recordings of activity in the secondary somatosensory cortex, performed significantly greater than chance at classifying pain and better than any of the other regions tested. Our findings are consistent with the secondary somatosensory cortex being the region which is most often reported to activate during painful stimulation [30], [33], [35]. Our findings are also consistent with the theory that one primary role of the secondary somatosensory cortex in pain perception is to discern whether stimuli are painful or non-painful [36]–[38]. However, accuracy of the SVM which was trained using only data from the secondary somatosensory cortex was below that of the whole-brain approach. Therefore, we find that pain assessment based on whole-brain BOLD patterns of activity performs better than assessment based on the activity in individual brain regions.

We further found that the accuracy of the SVM classifier could be enhanced by employing distance from the separating hyperplane as a measure of the classifier's confidence. Greater distance from the separating hyperplane was indicative of greater confidence in the SVM classification. By taking this information into account, each classification was associated with a probability of its accuracy, and the SVM classifier's overall accuracy was increased, at the cost of excluding some stimuli on the basis of ambiguity.

While this study was designed to probe the use of physiology-based pain detection, the results also more largely suggest that the brain's neural representation of pain is robust and replicable across individuals. We found that the SVM classifier performed more accurately than chance when applied to study participants in both the test group and the retest group. This finding shows that across individuals, pain-induced BOLD signal changes are considerably similar with regard to both spatial location and absolute magnitude, measured in units of percent BOLD signal change. Therefore, while there may be considerable individual differences in the experience of pain and in patterns of brain activity induced by pain, there are nonetheless a core set of pain-induced responses in the brain that may be universal, at least when considering discreet thermal pain stimuli.

We are still very far from a physiology-based pain assessment tool that could be used in clinical, forensic, and other applied settings. However, we see the goal of an accurate, valid surrogate for self-reported pain as both attainable and worthy of effort. There are several areas where the method reported here for detecting pain can be improved. We outline five specific tasks below.

First, supervised machine learning algorithms should be used in conjunction with fMRI to extend the approach reported here by investigating pain intensity, and by distinguishing brain activity related to stimulus intensity from brain activity related to pain intensity. The potential of using fMRI and machine learning algorithms to measure pain intensity has been demonstrated using a within-person analysis [18], yet it remains unknown whether the approach can provide accurate measurements when data from the test subject are not included in training. At minimum, using an approach similar to the one used here, classifiers should be able to distinguish between low, moderate, and high levels of pain.

Second, using fMRI and machine learning algorithms, future experiments should develop physiology-based pain assessments that perform accurately across sensory modalities. While recent work has demonstrated that a major component of the brain regions activated by pain are also activated by non-painful somatosensory stimuli [39], we show here that painful stimuli can be distinguished from non-painful stimuli by the magnitude of activation; others have shown that painful stimuli can be distinguished from nonpainful stimuli by the time course of activation [40], [41]. Taking into account the spatial location of brain activity, its magnitude, and its change over time, future studies should identify patterns of brain activity that distinguish pain regardless of the causal stimulus (for example, thermal, electrical, and mechanical stimuli should be tested). Doing so would further elucidate neural mechanisms that distinguish pain processing from sense modality-specific processing. The stimuli would also need to be tested in various locations on the body, to avoid developing models that are only accurate at assessing pain evoked in a specific body region.

Third, supervised machine learning algorithms should be developed that can distinguish pain from affective conditions that induce patterns of brain activity that are similar to those induced by pain. While previous research suggests that many of the brain regions that were most involved in driving the SVM's performance are associated with the sensory dimensions of pain such as pain intensity and localization [30], [33], [35], it is necessary to ensure that the approach used here is both sensitive and specific to pain. Therefore, a series of experiments should be conducted to determine whether SVM models can accurately distinguish physical pain from related affective experiences such as anticipation of pain [42], pain empathy [43], imagined pain [44], and social exclusion [45]. These experiments would further validate the use of fMRI and machine learning algorithms as an approach which is not only accurate in controlled experimental settings, but in applied settings as well.

Fourth, SVM accuracy at classifying pain should be increased by incorporating various physiological and trait-based measurements. Sources of physiologic information such as skin conductance [10], heart rate [8], and pupil dilation [46] have been shown to correlate with measurements of pain. Similarly, trait differences such as gender [47], [48], genotype [49], fear of pain [50], and pain catastrophizing [51] have also been shown to correlate with measurements of pain. SVM and related machine learning algorithms are versatile tools that can learn complex relationships between multiple inputs; therefore, they are well suited for integrating varied measurements to make classifications which are more accurate than would result from the investigation of one data source in isolation. The goal in utilizing diverse data streams would be to yield accuracy levels as close to 100% as possible.

Fifth, future experiments should develop fMRI-based machine learning algorithms that can measure chronic pain. We have shown here that it is feasible to classify transient pain experiences by comparing the period of stimulation to a preceding pain-free rest period. While this is a major development, the method does not easily translate to chronic pain assessment because in patients with chronic pain, it is difficult to obtain a pain-free rest condition. More complex measurements of brain activity, for example, temporal covariance of the activity between regions, have been shown to correlate with pain perception [52]. These methods should be used in conjunction with supervised machine learning algorithms to provide greater information and to generate physiology-based models that perform accurately at detecting chronic pain.

There are many machine learning algorithms and thus, many alternatives to SVM classification when using multi voxel pattern analysis and fMRI data to assess pain. As we have done here, other groups have used linear classifiers, such as SVMs and Fisher's linear discriminant, to distinguish two or more cognitive states using patterns of brain activity [18], [28], [53]. In the case of fMRI data, there are typically many more features than examples, and therefore, one advantage of linear classifiers is a reduced risk of over-fitting. Furthermore, the direct comparison of linear and nonlinear classifiers of fMRI data has not demonstrated any advantage in accuracy when using a nonlinear classifier [53]. Thus the greater model simplicity makes linear classifiers an attractive option. One alternative to SVM classification is the use of Gaussian process models, which are well suited for probabilistic classification, in which a machine learning algorithm not only classifies but also provides a measure of probability for each example belonging to a particular class. Marquand et al. have compared this method to SVM classification and find that accuracies are similar [18]. Other approaches such as Gaussian process regression may be useful for measuring continuous variables such as pain intensity [18].

In conclusion, without relying on self-report from tested subjects, we demonstrate that in a controlled experimental setting, whole-brain patterns of brain activity can be used to assess whether a heat stimulus is painful. The results suggest that to advance the development of a physiology-based pain measure, neuroimaging methods can benefit from incorporating machine learning techniques, and from deeper investigation of the complex interplay of brain regions in mediating the experience of pain.
